# Supplementary material for: Mechanisms governing the pioneering and redistribution capabilities of the non-classical pioneer PU.1
Source: Nat Commun. 2020 Jan 21;11:402. doi: 10.1038/s41467-019-13960-2 (PMC6972792; doi:10.1038/s41467-019-13960-2)
Supplement: Supplementary file 7 — Source data [file 41467_2019_13960_MOESM7_ESM.zip › Source_Data/Figure6/Figure6B_MotifScanOutput/FLI1_PU.1/homerResults/motif7.similar.html]

motif7

## Information for motif7

A
G
T
C
G
A
C
T
A
G
C
T
A
G
T
C
G
A
C
T
A
G
C
T
A
G
T
C
G
A
C
T
A
G
C
T
A
G
T
C
G
A
C
T
A
G
C
T
A
G
T
C
  
Reverse Opposite:  

T
C
A
G
T
C
G
A
C
T
G
A
T
C
A
G
T
C
G
A
C
T
G
A
T
C
A
G
T
C
G
A
C
T
G
A
T
C
A
G
T
C
G
A
C
T
G
A
T
C
A
G
  

|  |  |
| --- | --- |
| p-value: | 1e-207 |
| log p-value: | -4.776e+02 |
| Information Content per bp: | 1.648 |
| Number of Target Sequences with motif | 4999.0 |
| Percentage of Target Sequences with motif | 50.25% |
| Number of Background Sequences with motif | 12129.4 |
| Percentage of Background Sequences with motif | 33.24% |
| Average Position of motif in Targets | 100.5 +/- 50.1bp |
| Average Position of motif in Background | 99.2 +/- 85.7bp |
| Strand Bias (log2 ratio + to - strand density) | -0.1 |
| Multiplicity (# of sites on avg that occur together) | 1.67 |
| Motif File: | file (matrix) reverse opposite |

### Similar de novo motifs found

|  |  |  |  |  |  |  |  |
| --- | --- | --- | --- | --- | --- | --- | --- |
| Rank | Match Score | Redundant Motif | P-value | log P-value | % of Targets | % of Background | Motif file |
| 1 | 0.951 | A G C T A G T C G A C T A G C T A G T C G A C T A G C T A G T C A G C T G A C T A G T C A G C T G A C T A G T C | 1e-131 | -303.300525 | 55.72% | 41.93% | motif file (matrix) |
| 2 | 0.757 | G T C A C G T A C T A G G T C A G C T A C T A G G T C A G C T A T A C G C G T A A C T G C T A G G C T A C T G A | 1e-90 | -209.488845 | 11.41% | 5.33% | motif file (matrix) |
| 3 | 0.824 | A C T G C T G A T C G A C T A G T C G A G C T A A T C G T G C A C T G A T C A G T C G A | 1e-43 | -101.027556 | 3.09% | 1.01% | motif file (matrix) |
| 4 | 0.882 | A G T C G A C T G A C T G T A C C G A T A G C T G T A C A C G T A G C T A G T C G A C T | 1e-32 | -75.037083 | 2.00% | 0.59% | motif file (matrix) |
